# Temporalis muscle biomarkers from routine brain MRI and risk of dementia in two independent cohorts

**DOI:** 10.1002/alz.71622

**Published:** 2026-08-06

**Authors:** Kamyar Moradi, Roham Hadidchi, Amyn Majbri, Timothy M. Hughes, Hanzhang Lu, Yuxin Zhu, Soheil Mohammadi, Sara Momtazmanesh, Pratik Mukherjee, Muhammad Abdullah, Eleanor Simonsick, Jennifer A. Schrack, Marcus D. Goncalves, Josef Coresh, Marilyn Albert, Shadpour Demehri

**Affiliations:** ^1^ Russell H. Morgan Department of Radiology and Radiological Science Johns Hopkins University School of Medicine Baltimore Maryland USA; ^2^ Optimal Aging Institute Department of Population Health New York University Grossman School of Medicine New York New York USA; ^3^ Wake Forest University School of Medicine Wake Forest University Winston‐Salem North Carolina USA; ^4^ Department of Biostatistics Johns Hopkins Bloomberg School of Public Health Baltimore Maryland USA; ^5^ Mallinckrodt Institute of Radiology Washington University in St. Louis Saint Louis Missouri USA; ^6^ Department of Radiology University of California San Francisco San Francisco California USA; ^7^ Translational Gerontology Branch National Institute on Aging Intramural Research Program Baltimore Maryland USA; ^8^ Department of Epidemiology Johns Hopkins Bloomberg School of Public Health Baltimore Maryland USA; ^9^ Department of Neurology Johns Hopkins University School of Medicine Baltimore Maryland USA

**Keywords:** brain MRI, dementia, muscle size, temporalis muscle

## Abstract

**INTRODUCTION:**

Skeletal muscle loss is associated with cognitive decline, but whether neuroimaging‐derived muscle characteristics predict incident dementia remains unclear.

**METHODS:**

We evaluated associations of deep learning–derived temporalis muscle (TM) cross‐sectional area (CSA) and radiomic texture features from baseline T1‐weighted magnetic resonance imaging (MRI) with incident dementia in dementia‐free participants from the Alzheimer's Disease Neuroimaging Initiative (ADNI) (*n* = 750) and the Atherosclerosis Risk in Communities (ARIC) study (*n* = 532). TM was segmented using a convolutional neural network trained in ADNI and externally validated in ARIC. Radiomic features were reduced using least absolute shrinkage and selection operator‐penalized Cox models to generate a composite score. Multivariable Cox regression adjusted for demographics, *apolipoprotein E* ε4, baseline cognition, body mass index, and physical performance.

**RESULTS:**

Higher TM radiomic scores were associated with increased dementia risk in ADNI (hazard ratio per SD, 1.32) and ARIC (1.64). Smaller TM CSA predicted dementia in ADNI but not ARIC.

**DISCUSSION:**

TM texture patterns from routine brain MRI are associated with dementia risk, supporting TM phenotyping as a scalable marker of systemic biological vulnerability.

## Highlights

- TM radiomic texture patterns from routine brain MRI predict incident dementia across two cohorts.
- Cross‐sectional area shows weaker and cohort‐dependent associations with dementia risk.
- Opportunistic imaging of muscle on brain MRI may capture systemic biological vulnerability to dementia.

## 1 BACKGROUND

Dementia incidence increases rapidly with advancing age.^1^ Although disease‐modifying therapies targeting amyloid pathology have demonstrated substantial target engagement, their effects on slowing cognitive decline and disease progression remain modest. Accordingly, there is continued interest in identifying clinically meaningful markers of vulnerability that may inform risk stratification, prognosis, and preventive strategies earlier in the disease course.^2^, ^3^ Dementia has been consistently observed to co‐occur with skeletal muscle loss,^4^, ^5^, ^6^ but this association is potentially confounded by physical inactivity, poor nutrition, cerebrovascular and metabolic dysfunction, systemic inflammation, and other factors that contribute to overall biological vulnerability, including frailty, low educational attainment, genetic predisposition (e.g., apolipoprotein E [*APOE]* ε4 carrier status), and comorbid medical conditions, all of which contribute to both muscle loss and dementia risk.^7^ A meta‐analysis of 15 cross‐sectional studies reported that skeletal muscle loss was associated with a 2.25‐fold increased likelihood of concurrent cognitive impairment.^8^ However, longitudinal evidence establishing the temporal relationship between low muscle mass and dementia risk is lacking. If reductions in muscle size occur prior to overt cognitive decline, and if interventions that preserve or restore muscle health favorably influence cognitive trajectories and function, then early detection of skeletal muscle loss may offer a window for risk stratification and preventive intervention.^9^, ^10^, ^11^, ^12^, ^13^

In this context, a widely available and tractable imaging biomarker of skeletal muscle size could facilitate early skeletal muscle loss detection and be used to investigate the potential benefits of modifying interventions (e.g., resistance training, aerobic exercise, nutritional interventions) in mitigating dementia risk and progression. Traditional methods of assessing muscle health, such as clinical assessments,^14^ anthropometry,^15^ dynamometry, and serum/urine markers,^16^ have limited sensitivity, and more importantly are not regularly measured in prospective dementia cohort studies (or clinically).^17^, ^18^ Current guidelines demonstrate a growing trend towards imaging biomarkers of muscle composition. Dual‐energy X‐ray absorptiometry (DEXA) is recommended as “standard of care” in clinical practice,^19^ but is limited by lack of adherence and cost, and is not included in cognitive research studies or clinical evaluation for dementia.^20^

During initial cognitive evaluations in older adults, brain MRI is frequently ordered to evaluate for potential neuroanatomic changes. Beyond its role in evaluating neuroanatomy, brain MRI also captures the temporalis muscle (TM), which is anatomically stable, well‐bordered, and easy to segment. As the largest cranial skeletal muscle, the TM's size has been found to correlate with general body skeletal muscle mass,^21^, ^22^, ^23^ providing a unique opportunity to assess overall skeletal muscle health from existing brain MRI data that may otherwise go overlooked. Numerous previous studies showed that TM size predicted outcomes in head and neck cancers,^24^, ^25^, ^26^ strokes,^27^, ^28^, ^29^ Parkinson's disease,^30^ and amyotrophic lateral sclerosis^31^ (eIntroduction in Supplement 1). Cross‐sectional findings also suggest correlations between TM thickness, appendicular muscle mass, and cognitive function.^21^, ^23^, ^32^, ^33^, ^34^, ^35^, ^36^ However, the TM's longitudinal predictive value for dementia risk has yet to be evaluated.

In this study, we used data from the Alzheimer's Disease Neuroimaging Initiative (ADNI) and Atherosclerosis Risk in Communities (ARIC) cohorts to examine whether baseline TM cross‐sectional area (CSA), as well as radiomic features, measured opportunistically from routine brain MRIs, are independently associated with and predict subsequent dementia risk. We hypothesized that TM characteristics may serve as an integrated indicator of overall biological vulnerability to dementia risk, capturing the cumulative influence of underlying frailty, systemic health, nutrition, and comorbid medical conditions. By leveraging these routinely available imaging features, we aimed to evaluate whether this integrated measure of multisystem vulnerability provides prognostic information beyond traditional established demographic, clinical, and genetic risk factors.

## 2 METHODS

### 2.1 Study design and participants

**RESEARCH IN CONTEXT**

1. **Systematic review**: Reviewing the literature in public databases and search engines, we found studies reporting that 1D TM thickness correlates with general body skeletal muscle size and predicts outcomes across various neurological conditions (eIntroduction in Supplement 1). However, we found no longitudinal studies using 2D or high‐dimensional TM phenotyping from routine brain MRI (i.e., cross‐sectional area and radiomic texture patterns) to evaluate risk of incident dementia in initially dementia‐free participants or studies demonstrating replication of such imaging‐derived measures across independent cohorts.
2. **Interpretation**: In this longitudinal cohort study of 1282 dementia‐free participants from the ADNI and the ARIC Neurocognitive Study, altered TM texture patterns from T1‐weighted brain MRI were consistently associated with a higher risk of incident dementia over 7 to 10 years of follow‐up across both cohorts. Smaller TM cross‐sectional area was modestly associated with dementia risk in one cohort but not the other, possibly due to demographic heterogeneity between the cohorts.
3. **Future directions**: Future studies should replicate these findings in more diverse cohorts and explicitly test subgroup performance to improve generalizability and mitigate bias. Methodological work should evaluate volumetric (not single‐slice) temporalis measures, multi‐sequence MRI for muscle composition, and cross‐site harmonization to improve robustness. Prospective studies should test whether reporting TM metrics from routine brain MRI can trigger targeted exercise/nutrition interventions and whether this changes downstream cognitive and functional trajectories.

Data utilized for this observational cohort study were sourced from two independent observational cohorts: the ADNI and the ARIC Neurocognitive Study (ARIC‐NCS), two cohorts that were initiated in 2003 and 1987, respectively. ADNI included individuals aged 55 to 90 years classified as cognitively normal or as having mild cognitive impairment or dementia. Persons with significant systemic illness, unstable medical conditions, major depression, or baseline brain imaging showing focal lesions or multiple lacunae were excluded from ADNI's cohort.^37^ Eligibility criteria for ARIC‐NCS encompassed adults aged 45 to 64 years at baseline, with longitudinal neurocognitive follow‐up beginning in midlife. Within ARIC‐NCS, participants with prior stroke, major neurologic disease, baseline dementia, or marked depressive symptoms (Center for Epidemiologic Studies Depression Scale > 8) were excluded during the study's neurocognitive adjudication process.^38^, ^39^

For the opportunistic measure of TM size using conventional brain MRI, we utilized baseline T1‐weighted brain MRIs from ADNI‐1 and ADNI‐Grand Opportunities (ADNI‐GO), including non‐demented participants. We also used data from participants with MCI at Visit 5 of the ARIC‐NCS. We included participants who had available T1‐magnetization‐prepared rapid gradient echo (T1‐MPRAGE) MRI sequences at the study baseline and at least one follow‐up cognitive adjudication. Baseline here is defined as ADNI‐1 or ADNI‐GO and Visit 5 of ARIC‐NCS. The following baseline (ADNI‐1 or ADNI‐GO and Visit 5 of ARIC‐NCS) demographic, genetic, cognitive, functional, and imaging variables were extracted for analysis: age, sex, race, body mass index (BMI), *APOE* ε4 carrier status, Mini‐Mental State Examination (MMSE) score, and Short Physical Performance Battery (SPPB, available only in ARIC‐NCS) score. Analyses were conducted using a complete‐case approach. Participants were required to have an available baseline T1‐weighted MRI, at least one follow‐up cognitive adjudication, and non‐missing data for all covariates included in the corresponding multivariable model; no imputation was performed.

### 2.2 MRI acquisition and TM measurement

**FIGURE 1:**
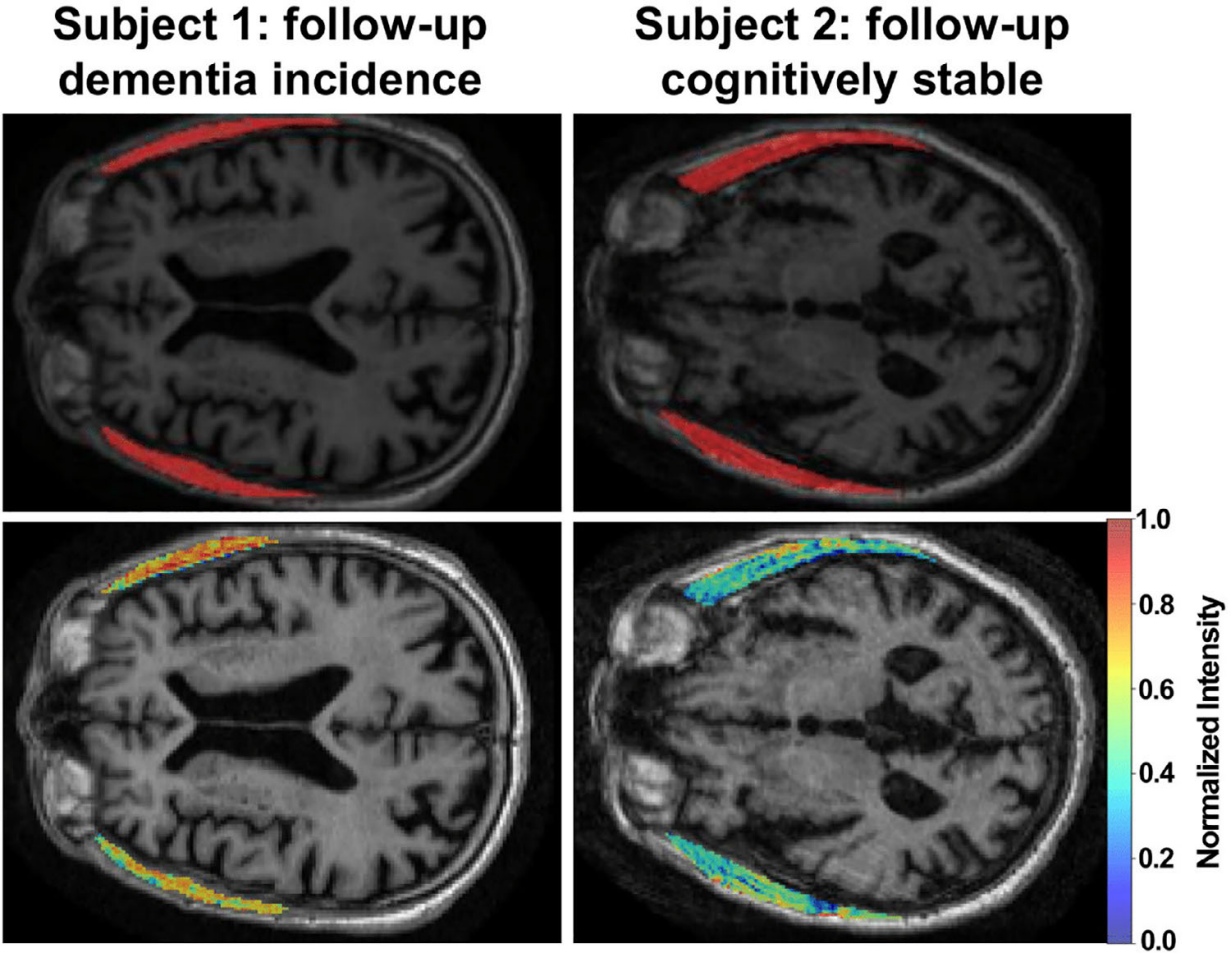
Temporalis muscle measurements and dementia outcomes in two participants from Alzheimer's Disease Neuroimaging Initiative (ADNI). Axial brain MRI scans of two demographically and clinically comparable participants at baseline (Subject 1: age: 85.5, sex: male, Mini‐Mental State Examination: 30, *APOE* ε4: absent; Subject 2: age: 84.8, sex: male, Mini‐Mental State Examination: 28, *APOE* ε4: absent). The top row shows a reduced cross‐sectional area (CSA, i.e., 2D size) of the temporalis muscle, typically anatomically stable, well‐bordered, and easy to segment, in Subject 1 (702 mm^2^ [32.2nd percentile in ADNI]) compared to Subject 2 (1444.2 mm^2^ [96.7th percentile in ADNI]), along with radiomic feature maps illustrating differences in tissue heterogeneity and lower mean intensity values in Subject 1 relative to Subject 2. During follow‐up, Subject 1 developed dementia over 8 years, whereas Subject 2 remained cognitively stable over 10 years of follow‐up.

TMs were manually segmented on axial T1‐weighted MRIs from 750 ADNI participants at the orbital‐roof level, with blinded dual readers and standardized preprocessing; full protocols and reproducibility metrics are detailed in eMethods (Supplement 1). An ImageNet‐pretrained ResNet‐50 U‐Net was trained on 80% of ADNI images, validated on the remaining 20%, externally validated on 278 randomly selected ARIC MRI scans, and used to generate all final deep learning‐derived TM masks for radiomic and CSA analyses (Dice similarity coefficient [DSC] reported). Representative cases from two ADNI participants are shown in Figure 1.

### 2.3 Outcomes

In ADNI, incident dementia was defined according to National Institute of Neurological and Communicative Disorders and Stroke (NINCDS‐ADRDA) criteria, operationalized as a Clinical Dementia Rating (CDR) global score ≥ 1.0, MMSE scores typically ranging from 20 to 26, impairment across multiple cognitive domains, and functional decline not attributable to other neurologic or psychiatric conditions, which corresponds to probable Alzheimer's disease.^17^ In the ARIC‐NCS (between Visit 5 and Visit 7 for up to 8 years of follow‐up), incident dementia was defined according to the Diagnostic and Statistical Manual of Mental Disorders, Third Edition, Revised and NINCDS‐ADRDA criteria, based on impairment in memory and at least one other domain (derived from domain‐specific z‐scores ≤ −1.5), corroborated by functional decline from the CDR scale and informant interviews, with final diagnoses adjudicated by a multidisciplinary committee, which corresponds to all‐cause dementia.^40^, ^41^ Etiologic dementia subtypes were not examined in this study because the ARIC endpoint reflects all‐cause dementia ascertainment and ADNI reflects clinically diagnosed probable Alzheimer's disease.

### 2.4 Radiomic feature extraction and dimensionality reduction

Using the PyRadiomics library, we extracted 93 radiomic features from the segmented TM of each participant.^42^, ^43^, ^44^, ^45^ These features spanned multiple domains, including first‐order intensity statistics (e.g., mean, variance, skewness), shape descriptors (e.g., compactness, elongation), and texture metrics derived from gray‐level co‐occurrence and run‐length matrices, capturing heterogeneity and structural complexity beyond conventional size measures.^42^, ^46^ To minimize overfitting and data leakage, feature selection and model training were performed independently within each cohort using cross‐validation, stability selection, and penalization. To address the high dimensionality of radiomic data and potential cohort‐specific variability, radiomic features were extracted using identical preprocessing parameters in both cohorts, and distributions were examined across scanners and sites. To reduce redundancy, we excluded highly correlated features within each cohort (Pearson *r* > 0.90). Remaining features were z‐standardized and used as predictors in a least absolute shrinkage and selection operator (LASSO)–penalized Cox proportional hazards model, along with age, sex, race, BMI, *APOE* ε4 carrier status, baseline MMSE, and additionally in ARIC, baseline SPPB. To further mitigate cross‐cohort differences in MRI protocols, LASSO models were trained independently within ADNI and ARIC, ensuring that radiomic features predictive in one cohort were not driven by differences in MRI protocol parameters between the two cohorts. This process yielded a sparse, stable subset of radiomic features with non‐zero coefficients. These features were then linearly combined, weighted by their LASSO‐derived coefficients, to generate a single composite radiomic score for each participant. Although models were trained independently, selected features overlapped across cohorts (Jaccard index = 0.43), demonstrating moderate to strong replicability of the radiomic signature.^47^, ^48^

Because high‐dimensional modeling can be prone to overfitting, data leakage, and biased feature selection,^49^ we implemented the following safeguards: (1) five‐fold cross‐validation to select the optimal penalization parameter (λ), balancing model fit and parsimony; (2) stability selection, in which feature inclusion was confirmed across resampled subsets, ensuring the robustness of selected predictors; (3) shrinkage via L1 penalization, which constrains coefficient magnitude and limits spurious associations; and (4) post‐selection inference checks, including examination of influential observations and assessment of proportional hazards assumptions via Schoenfeld residuals.^50^, ^51^, ^52^, ^53^ Cross‐validation results and stability‐selection metrics are provided in eFigure 1, eFigure S2, eFigure S3, and eTable S1 in eMethods (Supplement 1).

### 2.5 Statistical analysis

We evaluated the association between baseline TM CSA and composite radiomic score (continuous independent variables) and subsequent dementia incidence. After testing the assumptions of log‐linearity, proportional hazards, and presence of outlying or influential observations, we utilized a Cox proportional hazards model to measure the subsequent risk of dementia incidence (time‐to‐event outcome) per standard deviation (SD) decrement in TM CSA or SD change in radiomic score. To allow comparison with known dementia risk factors, continuous effect sizes were reported per SD change in the predictor derived separately in each cohort. To compare model predictive power with and without TM measures included as predictors, we applied paired bootstrap resampling (1000 iterations) to obtain concordance index (C‐index) estimates for each specification. The mean C‐index difference, along with its 95% confidence interval and two‐sided *p* value, was then derived from the resulting bootstrap distribution.^54^

To account for potential confounders, all models were adjusted for known risk factors of dementia outcomes including baseline age, sex, race, BMI, *APOE* ε4 carrier status, MMSE score, and, additionally in ARIC, SPPB score (for directed acyclic graph see eFigure S4, eMethods, Supplement 1). Categorical variables were compared using the χ^2^ test, and continuous variables were compared using the Wilcoxon rank‐sum test. A two‐tailed *p* value below 0.05 was considered statistically significant. Variables were also compared using standardized mean differences (SMDs), with values greater than 0.1 considered potentially meaningful. Statistical analyses were performed using R software version 4.0.3 and Python software version 3.11.

### 2.6 Sensitivity analysis

To test the robustness of this observational longitudinal analysis, we conducted an array‐based quantitative bias analysis to estimate the effect of residual confounding and quantify the extent to which such a confounder would need to differ across comparison groups, and be associated with the outcome, in order to fully explain the observed effect estimate.^55^ In addition, a subgroup analysis was performed, stratifying the analysis of each variable by baseline age, sex, race, BMI, *APOE* ε4 carrier status, MMSE score, and, in ARIC, SPPB score. In both ADNI and ARIC, we repeated the primary time‐to‐event analyses with two variations: (1) excluding participants who experienced dementia within the first 2 years of follow‐up and (2) using Fine‐Gray subdistribution hazards models treating all‐cause mortality as a competing event.^56^ In ARIC, we identified participants with baseline or incident competing neurological events during follow‐up, including stroke/cerebrovascular accident, head injury, Parkinson's disease, and brain tumor. We then repeated the analyses by (1) treating these neurological events, together with all‐cause mortality, as competing events in Fine‐Gray models and (2) excluding participants with baseline and/or incident neurological events in Cox proportional hazards models. In ARIC, we performed additional models adjusting for available dental/oral health variables collected at Visit 4, including loss of natural teeth, false teeth, root canal history, and dental implant history.

## 3 RESULTS

### 3.1 U‐Net segmentation model development

**FIGURE 2:**
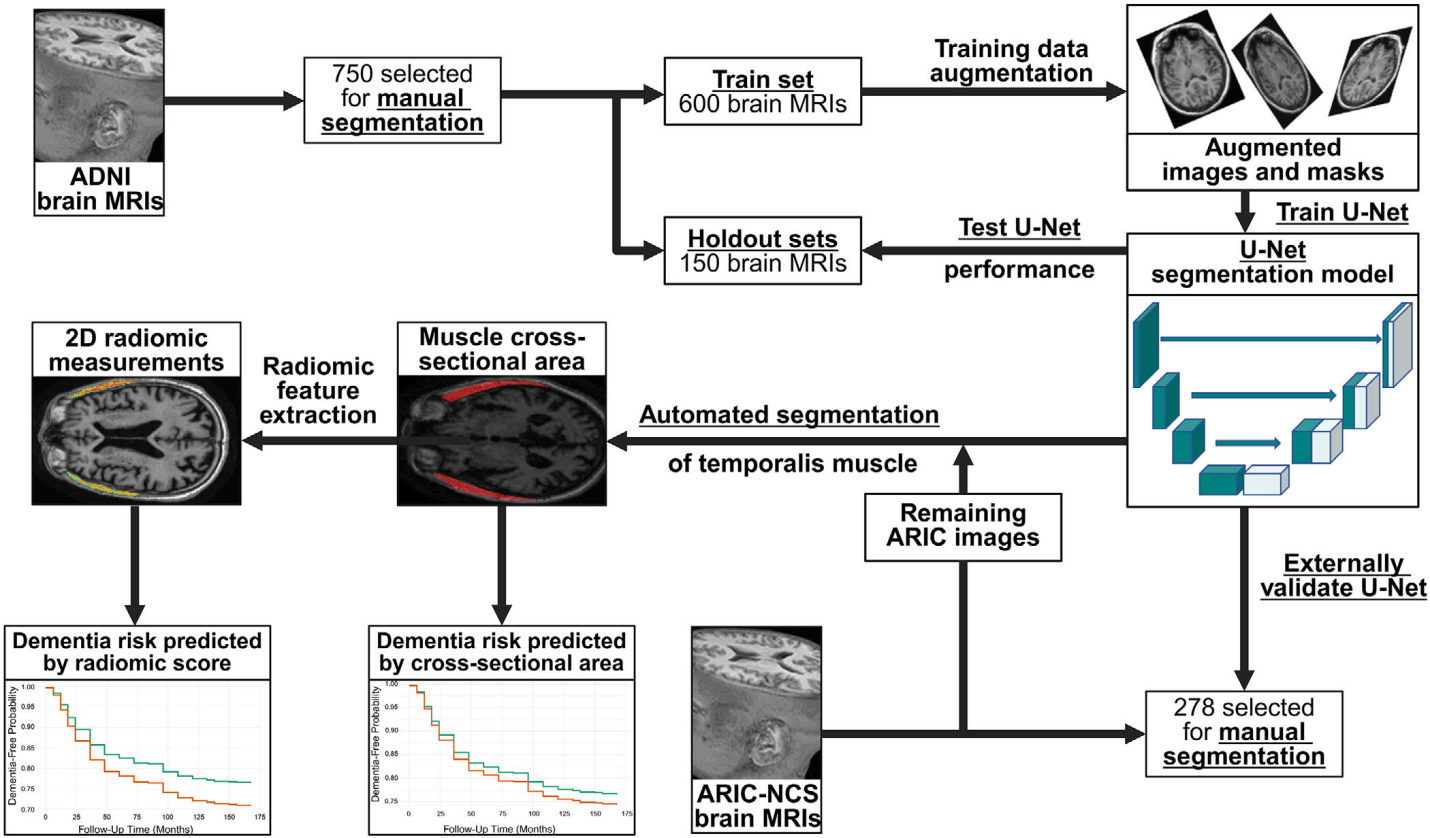
Workflow for temporalis muscle segmentation, feature extraction, and dementia risk prediction. From the Alzheimer's Disease Neuroimaging Initiative (ADNI), 750 T1‐weighted brain MRIs were manually segmented and split into a training set (*n* = 600) and a holdout test set (*n* = 150). Training data were augmented and used to train a U‐Net segmentation model, which was then tested on the holdout set. External validation of the U‐Net was performed on 278 manually segmented ARIC MRI scans. The model was then applied to MRI scans of participants from ADNI and Atherosclerosis Risk in Communities (ARIC) MRI scans, enabling automated temporalis muscle segmentation and extraction of cross‐sectional area and 93 radiomic features, which were combined into a radiomic score. Both cross‐sectional area and radiomic score were used in Cox proportional hazards models to predict incident dementia risk in both cohorts.

Figure 2 presents a schematic workflow for the study. The U‐Net segmentation model was trained on 80% of MRI scans in ADNI. The model achieved a DSC of 0.91 on the remaining 20% of ADNI images and 0.76 in 278 randomly selected MRI scans from ARIC.

### 3.2 Baseline characteristics

Table 1 summarizes baseline characteristics of 750 ADNI and 532 ARIC participants. Mean age was lower in ADNI (74.47 ± 7.04) than ARIC (76.80 ± 5.20) (*p* < 0.005, SMD = 0.37). The proportion of the cohort that was female was lower in ADNI (41.87%) than in ARIC (52.93%) (*p* < 0.005, SMD = 0.22). ARIC consisted of a markedly larger proportion of Black participants (24.23%) than ADNI (7.47%) (*p* < 0.005, SMD = 0.46). Baseline BMI was lower among ADNI participants (27.40 ± 4.55 vs 28.30 ± 5.40, *p* < 0.005, SMD = 0.18). ADNI participants were more likely than ARIC participants to be *APOE* ε4 carriers (42.27% vs 34.02%, *p* < 0.005, SMD = 0.17). Baseline MMSE was higher in ADNI (27.90 ± 1.80 vs 26.70 ± 2.60, *p* < 0.005, SMD = 0.55). These differences highlight the notable demographic and clinical heterogeneity between the two cohorts and the importance of validating imaging biomarkers across diverse populations.

**TABLE 1:** Baseline demographics of participants included in the analysis.

	ADNI (*n* = 750)	ARIC (*n* = 532)	*p* value	SMD
Age at baseline visit (years), mean ± SD	74.47 ± 7.04	76.80 ± 5.20	**<0.005**	0.37
Female, n (%)	314 (41.87%)	282 (52.93%)	**<0.005**	0.22
Body mass index (kg/m^2^), mean ± SD	27.40 ± 4.55	28.30 ± 5.40	**<0.005**	0.18
**Race**			**<0.005**	0.46
Non‐Hispanic White	694 (92.53%)	403 (75.75%)		
Non‐Hispanic Black	56 (7.47%)	129 (24.23%)		
*APOE* ε4 carrier, n (%)	317 (42.27%)	181 (34.02%)	**<0.005**	0.17
MMSE at baseline visit, mean ± SD	27.90 ± 1.80	26.70 ± 2.60	**<0.005**	0.55
Time from baseline to latest follow‐up (years), mean ± SD	10.31 ± 5.40	7.17 ± 2.73	**<0.005**	0.70
Incident dementia, n (%)	254 (33.87%)	126 (23.68%)	**<0.005**	0.23
SPPB at baseline visit, mean ± SD	Not available in ADNI	9.00 ± 2.50		

Abbreviations: ADNI, Alzheimer's Disease Neuroimaging Initiative; ARIC, Atherosclerosis Risk in Communities; CSA, cross‐sectional area; MMSE, Mini‐Mental State Examination; SD, standard deviation; SMD, Standardized Mean Difference; SPPB, Short Physical Performance Battery.

### 3.3 TM CSA and radiomic score and subsequent dementia incidence

Using continuous CSA data and Cox proportional hazards model fitting results, adjusted for baseline age, sex, race, BMI, *APOE* ε4 carrier status, and MMSE score, we found that a 1 SD decrement in the CSA of the TM was linked to a 16% increase in the risk of incident dementia (HR 1.16; 95% confidence interval [CI]: 1.01, 1.35; *p *= 0.042) (Table 2). In ARIC, the same adjusted model found no association between TM CSA and dementia risk (HR 1.05; 95% CI: 0.79, 1.41; *p* = 0.71). However, in ARIC, the association between TM CSA and dementia risk differed by race (*p* for interaction = 0.041), with a positive association among White participants (HR 1.32) but not Black participants (HR 0.77), who were more represented in ARIC than ADNI. Race interaction was not informative in ADNI subgroup analysis due to the small sample size of Black participants (*n* = 56) and wide confidence interval of HR (HR 1.02; 95% CI: 0.52, 2.00) (eFigure S5, eResults, Supplement 1).

**TABLE 2:** Cox proportional hazards models for incident dementia among participants in Alzheimer's Disease Neuroimaging Initiative (ADNI) and Atherosclerosis Risk in Communities (ARIC) cohorts.

	ADNI		ARIC	
Predictor	Hazard ratio [95% CI]	*p* value	Hazard ratio [95% CI]	*p* value
Temporalis muscle CSA (Z‐scored)	1.16 [1.01, 1.35]	**0.042**	1.05 [0.79, 1.41]	0.71
Temporalis muscle radiomic score (Z‐scored)	1.32 [1.12, 1.56]	**<0.005**	1.64 [1.26, 2.13]	**<0.005**
Age at baseline visit (Z‐scored)	1.00 [0.86, 1.07]	0.64	1.26 [1.00, 1.52]	**0.035**
Female versus male	0.96 [0.68, 1.36]	0.84	1.29 [0.80, 2.08]	0.29
Non‐Hispanic White versus non‐Hispanic Black	1.84 [1.01, 3.35]	**0.046**	0.90 [0.49, 1.63]	0.72
Body mass index (Z‐scored)	0.82 [0.71, 0.95]	**0.0080**	0.97 [0.75, 1.24]	0.79
*APOE* ε4 carrier	2.23 [1.72, 2.90]	**<0.005**	1.82 [1.27, 2.62]	**<0.005**
MMSE at baseline visit (Z‐scored)	0.64 [0.57, 0.72]	**<0.005**	0.70 [0.55, 0.89]	**<0.005**
SPPB at baseline visit (Z‐scored)	Not available in ADNI	−	1.07 [0.84, 1.36]	0.58

*Note*: Hazard ratios (HRs) for temporalis muscle cross‐sectional area (CSA) represent the risk of incident dementia per standard deviation decrease in CSA. HRs for the temporalis muscle radiomic score represent the risk of incident dementia per standard deviation increase in score. Ninety‐three radiomic features were used to derive a single radiomic score per participant using a least absolute shrinkage and selection operator regression model, which was trained independently in each cohort with five cross‐fold validation. Models were adjusted for baseline age, sex, race/ethnicity (non‐Hispanic White vs non‐Hispanic Black), *APOE* ε4 carrier status, body mass index, and Mini‐Mental State Examination. In ARIC, baseline Short Physical Performance Battery was additionally adjusted for.

Composite radiomic score was associated with dementia risk in both ADNI (HR per SD increase, 1.32; 95% CI: 1.12, 1.56; *p* < 0.005) and ARIC (HR 1.64; 95% CI: 1.26, 2.13; *p* < 0.005). Although radiomic score performance differed among Black participants in ADNI, visual quality control revealed no evidence of systematic misregistration; findings in this subgroup should be interpreted cautiously given the small sample size (*n* = 56). In ADNI, participants with a composite radiomic score below the mean had an incidence of 24.84 dementia cases per 1000 patient‐years, compared with 41.64 cases per 1000 patient‐years among those with scores above the mean. In ARIC, participants with a composite radiomic score below the mean had an incidence of 15.12 dementia cases per 1000 patient‐years, compared with 25.95 cases per 1000 patient‐years among those with scores above the mean. Kaplan‐Meier curves are shown in eFigure S6, eResults, Supplement 1. Incorporating TM radiomic measures into Cox proportional hazards demonstrated a modest improvement in model predictive power in the ADNI dataset (*p* value difference in concordance indices = 0.032) but not in the ARIC dataset (*p* value difference in concordance indices = 0.17) (eTable S2, eResults, Supplement 1).

The effect size of TM measures was modest relative to *APOE* ε4 and low MMSE, which were associated with a higher risk of incident dementia in both cohorts (*APOE* ε4 carrier status: ADNI HR 2.23, ARIC HR 1.82; lower baseline MMSE: ADNI HR 0.64, ARIC HR 0.70). However, the effect size of TM measures was higher than age, which was associated with a higher risk of dementia in ARIC (age: HR 1.26 per SD), and BMI, which was associated with a lower risk of dementia in ADNI (BMI: HR 0.82 per SD). In multivariate models in ARIC, SPPB was not associated with incident dementia risk (HR 1.07; 95% CI: 0.84, 1.36; *p* = 0.58) (Table 2).

### 3.4 Sensitivity analyses

**FIGURE 3:**
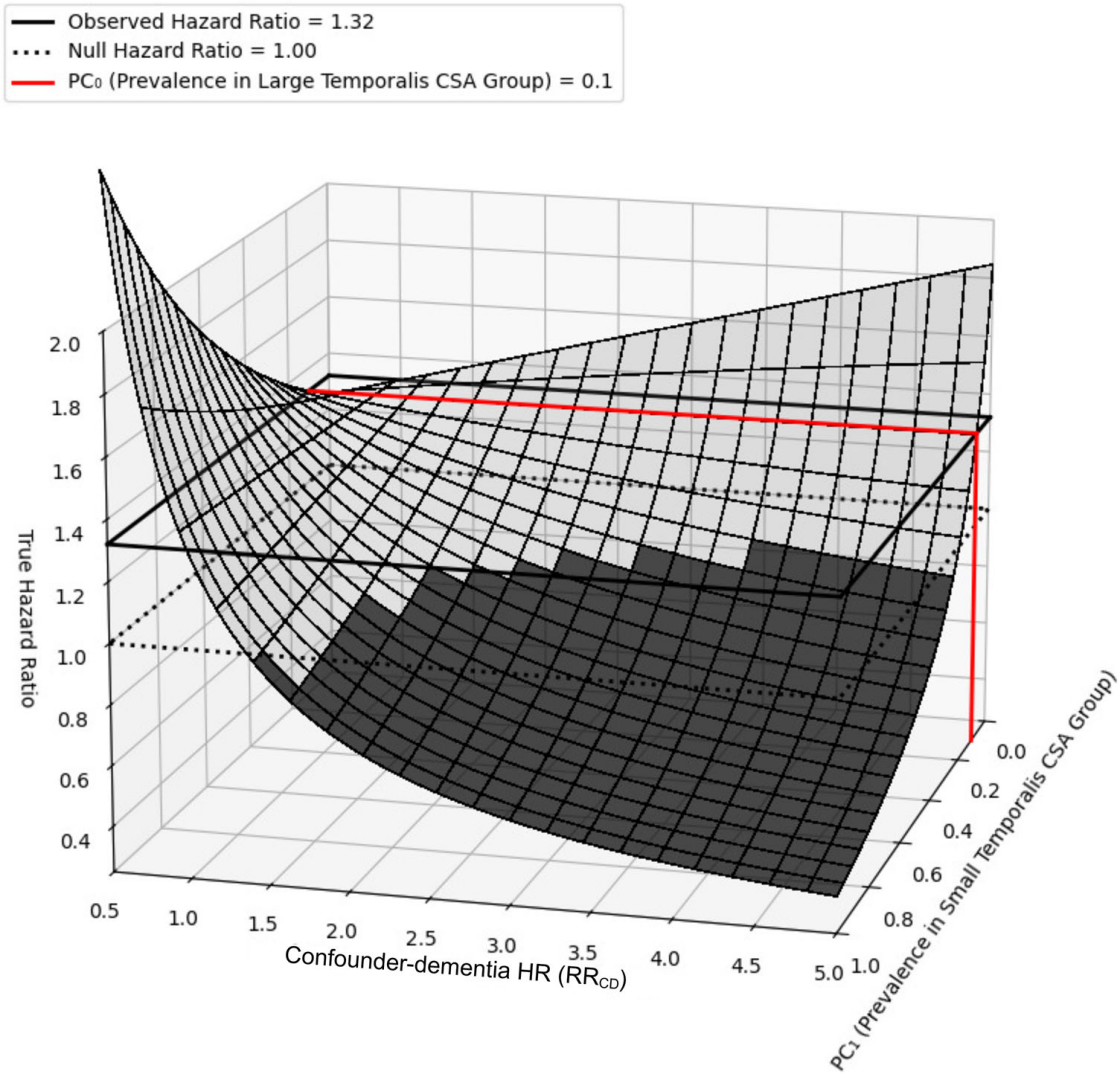
Sensitivity analysis evaluating the potential impact of residual confounding. The surface depicts the true hazard ratio, assuming an observed hazard ratio of 1.32 (between hypothetical large and small temporalis muscle groups) from the radiomics model in the Alzheimer's Disease Neuroimaging Initiative and a prevalence of the unmeasured confounder (PC0) of 0.1 (10%) in the large temporalis muscle group. RRCD represents the independent association between an unmeasured confounder and the risk of incident dementia. If a confounder had an independent risk ratio of 1.4 for incident dementia and was at least two times more prevalent in the small temporalis muscle group compared to the large temporalis muscle group, then the observed association could be nullified.

Physical performance, a critical confounder between skeletal muscle loss and dementia, was adjusted for in ARIC (via baseline SPPB score) but was not available in ADNI. Other confounders including physical activity levels and nutrition status were also not available. Thus, we performed an array‐based quantitative bias analysis to estimate the magnitude of residual confounding needed to nullify the observed association between TM radiomics and incident dementia (Figure 3). Given the observed HR of 1.32 (in ADNI), we found that if a hypothetical confounder had an independent risk ratio of 1.4 for incident dementia and was at least two times more prevalent in a hypothetical small TM group compared to a large TM group, then the observed association could be nullified.

Additionally, to verify the robustness of our results, several subgroup analyses were performed, stratifying by baseline age, sex, race, BMI, *APOE* ε4 carrier status, baseline MMSE score, and baseline SPPB score (in ARIC) (eFigure S5, eResults, Supplement 1).

As an additional sensitivity analysis, we excluded all participants who experienced dementia within the first 2 years of follow‐up and found consistent results (eTable S3, eResults, Supplement 1). In Fine‐Gray subdistribution hazards models treating all‐cause mortality as a competing event, the results were unchanged from the primary analyses (eTable S4, eResults, Supplement 1). In ARIC, competing neurological events such as stroke, head injury, Parkinson's disease, and brain tumor were relatively infrequent (eTable S5, eResults, Supplement 1); censoring them as competing events in Fine‐Gray subdistribution hazards models (eTable S6, eResults, Supplement 1) or excluding incident cases (eTable S7, eResults, Supplement 1) produced findings consistent with the primary analyses. In ARIC, dental and oral health factors were assessed at Visit 4 (eTable S8, eResults, Supplement 1), and additional adjustment for these factors did not alter the primary findings (eTable S9, eResults, Supplement 1).

## 4 DISCUSSION

In the longitudinal cohort studies of ADNI and ARIC, smaller TM CSA and higher composite radiomic scores, automatically measured from routine brain MRI, were independently associated with incident dementia over a follow‐up period of 7 to 10 years, with the radiomic association validated across these two cohorts with differing baseline risk distributions and demographic compositions. These findings suggest that TM phenotypes derived from routine neuroimaging may capture an imaging signature of biological vulnerability that precedes clinical dementia across heterogeneous populations.

### 4.1 TM as an integrated marker of biological vulnerability

Muscle loss and cognitive decline commonly co‐occur with aging and share multiple risk factors such as physical inactivity, poor nutrition, vascular and metabolic dysfunction, and systemic inflammation.^57^, ^58^ In our study, the association between TM measures and incident dementia persisted after multivariable adjustment for demographics, baseline BMI, MMSE, *APOE* ε4 carrier status, and, in ARIC, SPPB, with quantitative bias analysis suggesting that unmeasured confounding is unlikely to fully account for observed associations. Rather than implying a direct causal role, these findings support the interpretation of TM characteristics as an integrated marker of aging‐related biological vulnerability, reflecting cumulative vascular, inflammatory, and metabolic influences on dementia risk.

Critically, small muscle size, poor muscle quality, and their associated factors are modifiable. Multicomponent exercise programs reduce mobility disability and slow cognitive decline in high‐risk older adults, and targeted nutritional support further enhances these benefits.^59^, ^60^, ^61^, ^62^, ^63^ Yet persons most likely to benefit from these interventions are not systematically identified or referred.^64^, ^65^, ^66^ Existing diagnostic approaches frequently fail to detect early muscle loss: SARC‐F has poor sensitivity,^67^ BMI and calf circumference cannot detect sarcopenic obesity,^68^, ^69^ and performance‐based tools such as the SPPB require in‐person testing and do not quantify muscle structure,^70^ consistent with its non‐significant association with dementia risk in the ARIC multivariable model. DEXA and dedicated body‐composition CT/MRI are accurate but require separate imaging, cost, and resources and are rarely ordered for muscle assessment or in dementia risk evaluation.^71^, ^72^, ^73^ Since the TM is routinely captured on neuroimaging frequently ordered for the evaluation of cognitive decline^74^ and its size correlates with general body muscle size,^21^, ^23^, ^33^, ^34^, ^35^, ^36^ opportunistic assessment may help fill this gap, providing a zero‐burden, anatomically grounded, and scalable marker of systemic muscle health. Analogous opportunistic imaging strategies have demonstrated clinical impact: In the NOTIFY‐1 randomized trial, reporting coronary artery calcium scores to clinicians markedly increased preventive therapy use, demonstrating that surfacing imaging‐derived risk information could meaningfully change management.^75^ Although prospective validation is needed before clinical integration, TM measurement is safe, cost‐effective, and readily scalable and may similarly prompt earlier referral to exercise or nutritional interventions.

Although TM CSA was significantly associated with dementia risk in ADNI, but not in ARIC, our stratified analyses found it to be less predictive of dementia risk among Black participants than in White participants, which may partially explain the null ARIC finding given that cohort's higher proportion of Black participants. Prior work reported that muscle quality declines, such as fat infiltration, occur more prominently with aging among Black individuals despite higher absolute muscle mass compared with White individuals^76^, ^77^ and that the relationship between intermuscular adipose tissue and cognitive decline may itself differ by race.^78^ Notably, the radiomic score showed stronger associations with dementia risk among Black participants than among White participants, further supporting race‐based variation in how imaging‐derived muscle phenotypes relate to cognitive decline, though these findings warrant cautious interpretation. Methodological and clinical differences between cohorts may also contribute. CSA is a single‐slice geometric measure sensitive to segmentation and slice‐selection variability; the lower DSC in ARIC (0.76 vs 0.91 in ADNI) suggests greater measurement error, which would attenuate associations toward the null. Furthermore, muscle‐related phenotypes may align more closely with Alzheimer's disease–type neurodegeneration than with etiologically heterogeneous dementia syndromes: A recent meta‐analysis reported a stronger association between sarcopenia and Alzheimer's disease than with non–Alzheimer's disease dementias.^79^ Differences in outcome definition between ADNI (clinically probable Alzheimer's dementia) and ARIC (all‐cause dementia ascertainment) and their differing etiologic mix could therefore plausibly dilute size‐based associations in settings where the dementia endpoint is broader.

By contrast, analyses across both cohorts have consistently demonstrated the predictive relevance of muscle texture features, as captured by the composite radiomic score. Radiomic approaches have been applied to skeletal muscle,^80^, ^81^, ^82^, ^83^, ^84^ with texture features on T1‐weighted images shown to correlate with quantitative fat fraction measurements and detect early intramuscular fat accumulation. Elevated T1 values have further been linked to early inflammatory changes and later fibrotic remodeling, reflected by fibrotic markers such as α‐smooth muscle actin and fibronectin.^83^, ^84^ At the same time, individual radiomic features are agnostic by design, quantifying complex spatial and statistical properties of tissue signal rather than discrete biological processes. Drawing on 93 candidate features extracted per participant, the composite score is not biologically interpretable a priori but supports the concept that TM texture captures an imaging signature of biological vulnerability relevant to dementia risk. Accordingly, we interpret a 1‐SD increase in the radiomic score as a standardized shift toward this higher‐risk muscle microstructure pattern, rather than a direct mechanistic biomarker.

Overfitting is an important consideration in radiomics, particularly when many correlated features are analyzed relative to a limited number of events. To reduce this risk, we applied LASSO‐penalized feature selection, five‐fold cross‐validation for parameter tuning, and independent training within each cohort, with consistency examined across two cohorts and multiple sensitivity analyses. Further external validation in additional datasets remains warranted, and future work correlating the TM radiomic signature with quantitative fat fraction imaging (e.g., Dixon)^85^ and/or histologic correlates will be needed to clarify the underlying tissue characteristics it reflects.

The link between poor muscle health and dementia risk may be partially mediated by systemic inflammation and serum myokines, bioactive peptides secreted by skeletal muscles that cross the blood‐brain barrier and influence neuroplasticity, neuronal survival, and hippocampal function.^86^, ^87^, ^88^ However, it remains unclear whether TM characteristics reflect a causal role in cognitive decline or serve as an integrated marker of broader systemic aging, given shared behavioral and metabolic risk factors such as physical inactivity.^86^, ^89^, ^90^ Covariate adjustment and quantitative bias analysis support the persistence of the association beyond measured confounding, but future studies incorporating detailed lifestyle and activity data are needed to clarify the directionality of this relationship.

### 4.2 Limitations

Several limitations warrant consideration. First, the incremental improvement in model discrimination after adding TM measures was modest and cohort‐dependent, consistent with prior work on systemic and aging‐related biomarkers,^91^ implying that these measures are best interpreted as markers of biological vulnerability rather than tools for individual‐level dementia prediction. Second, we used a single axial MRI slice to assess TM CSA, an established approach that enables high‐throughput analysis of muscle size^92^ and texture^93^, ^94^ but may sacrifice granularity relative to volumetric assessment; future deep learning‐based volumetric approaches could improve replicability and predictive power. Third, radiomic features are agnostic by design and should be interpreted as an imaging signature of muscle microstructure and systemic vulnerability, found to correspond to dementia risk rather than a direct mechanistic biomarker. Fourth, we employed T1‐weighted MRIs for TM segmentation, as this sequence is consistently included in brain MRI protocols and has been successfully used for skeletal muscle segmentations and compositional analysis,^95^ though other sequences may improve compositional characterization. Fifth, adjustment for baseline MMSE may attenuate association estimates given its contribution to dementia adjudication, though we prioritized this adjustment to reduce confounding by baseline cognitive status. Sixth, only baseline TM characteristics were evaluated, precluding assessment of whether longitudinal changes in TM size or texture track cognitive decline, precede conversion to dementia, or provide incremental prognostic value beyond baseline measures. Future studies should characterize TM trajectories and test whether rates of change in TM characteristics correlate with cognitive decline and dementia risk. Seventh, racial diversity was limited in ADNI (7.47% Black vs 24.23% in ARIC), reducing statistical power for subgroup and interaction analyses and limiting generalizability of race‐stratified findings, which should be interpreted cautiously and considered exploratory rather than definitive. Further validation in larger, multi‐ethnic cohorts with broader sociodemographic representation will be important to determine the robustness and external validity of these associations. Eighth, dementia endpoints differed across cohorts, with ADNI capturing clinically diagnosed probable Alzheimer's disease and ARIC capturing adjudicated all‐cause dementia without reliable etiologic subtype data, potentially attenuating associations if TM phenotypes relate more strongly to Alzheimer's disease neurodegeneration than to other dementia etiologies. Future studies with subtype‐specific dementia adjudication will be important to determine whether these associations differ across Alzheimer's disease, vascular dementia, Lewy body dementia, and mixed etiologies. Ninth, the TM is a muscle of mastication, and its characteristics may be influenced by dental or chewing‑related factors, which may influence overall health status. While the only available dental health variables in ARIC were collected 13 to 17 years before Visit 5, adjusting for these variables had no tangible effect on the results. Future studies with contemporaneous oral health data would be useful. Lastly, while TM CSA correlates with generalized skeletal muscle mass,^21^, ^22^, ^23^ further validation using whole‐body imaging and diverse datasets is needed to confirm its utility as a surrogate for generalized skeletal muscle health.

## 5 CONCLUSIONS

TM size and compositional features, accessible on standard brain MRI, are independently associated with future dementia risk across two cohorts. These measures likely reflect integrated biological vulnerability, including systemic aging, frailty, and nutritional status, rather than disease‐specific processes, supporting their value as practical, low‐cost, radiation‐free, and scalable biomarkers deployable within existing neuroimaging workflows. Further research is needed to validate their clinical utility and determine whether monitoring or improving muscle health can alter dementia trajectories.

## CONFLICT OF INTEREST STATEMENT

The authors declare no conflicts of interest. Author disclosures are available in the Supporting Information.

## CONSENT STATEMENT

Written informed consent was obtained from all participants at enrollment in the Alzheimer's Disease Neuroimaging Initiative and the Atherosclerosis Risk in Communities Neurocognitive Study. This study involved secondary analysis of de‐identified data and was conducted in accordance with institutional and federal guidelines; no additional informed consent was required.

## Supporting information




Supporting Information

Supporting Information
