# Supplementary material for: Temporalis muscle biomarkers from routine brain MRI and risk of dementia in two independent cohorts
Source: Alzheimers Dement. 2026 Aug 6;22(8):e71622. doi: 10.1002/alz.71622 (PMC13445788; doi:10.1002/alz.71622)
Supplement: Supplementary file 2 [file ALZ-22-e71622-s001.docx]

# Supplemental Introduction

## 1.1. List of research studies examining potential predictive value of temporalis muscle biomarkers for clinical outcomes related to various neurological diseases

1.	Lee B, Bae YJ, Jeong W-J, Kim H, Choi BS, Kim JH. Temporalis muscle thickness as an indicator of sarcopenia predicts progression-free survival in head and neck squamous cell carcinoma. Scientific Reports. 2021;11(1):19717.

2.	Furtner J, Berghoff AS, Schöpf V, et al. Temporal muscle thickness is an independent prognostic marker in melanoma patients with newly diagnosed brain metastases. Journal of neuro-oncology. 2018;140(1):173-8.

3.	Furtner J, Weller M, Weber M, et al. Temporal Muscle Thickness as a Prognostic Marker in Patients with Newly Diagnosed Glioblastoma: Translational Imaging Analysis of the CENTRIC EORTC 26071-22072 and CORE Trials. Clinical cancer research : an official journal of the American Association for Cancer Research. 2022;28(1):129-36.

4.	Furtner J, Berghoff AS, Albtoush OM, et al. Survival prediction using temporal muscle thickness measurements on cranial magnetic resonance images in patients with newly diagnosed brain metastases. European radiology. 2017;27(8):3167-73.

5.	Sadhwani N, Aggarwal A, Mishra A, Garg K. Temporal muscle thickness as an independent prognostic marker in glioblastoma patients-a systematic review and meta-analysis. Neurosurgical review. 2022;45(6):3619-28.

6.	Muglia R, Simonelli M, Pessina F, et al. Prognostic relevance of temporal muscle thickness as a marker of sarcopenia in patients with glioblastoma at diagnosis. European radiology. 2021;31(6):4079-86.

7.	An G, Ahn S, Park JS, Jeun SS, Hong YK. Association between temporal muscle thickness and clinical outcomes in patients with newly diagnosed glioblastoma. Journal of cancer research and clinical oncology. 2021;147(3):901-9.

8.	Ten Cate C, Huijs SMH, Willemsen ACH, et al. Correlation of reduced temporal muscle thickness and systemic muscle loss in newly diagnosed glioblastoma patients. Journal of neuro-oncology. 2022;160(3):611-8.

9.	Liu F, Xing D, Zha Y, et al. Predictive Value of Temporal Muscle Thickness Measurements on Cranial Magnetic Resonance Images in the Prognosis of Patients With Primary Glioblastoma. Frontiers in neurology. 2020;11:523292.

10.	Furtner J, Genbrugge E, Gorlia T, et al. Temporal muscle thickness is an independent prognostic marker in patients with progressive glioblastoma: translational imaging analysis of the EORTC 26101 trial. Neuro-oncology. 2019;21(12):1587-94.

11.	Kim YI, Shin JY, Yang SH, Kim HH, Shim BY, Ahn S. Association between Temporal Muscle Thickness and Overall Survival in Non-Small Cell Lung Cancer Patients with Brain Metastasis. Current oncology (Toronto, Ont). 2022;29(9):6463-71.

12.	Nozoe M, Kubo H, Kanai M, et al. Reliability and validity of measuring temporal muscle thickness as the evaluation of sarcopenia risk and the relationship with functional outcome in older patients with acute stroke. Clinical neurology and neurosurgery. 2021;201:106444.

13.	Katsuki M, Yamamoto Y, Uchiyama T, Wada N, Kakizawa Y. Clinical characteristics of aneurysmal subarachnoid hemorrhage in the elderly over 75; would temporal muscle be a potential prognostic factor as an indicator of sarcopenia? Clinical neurology and neurosurgery. 2019;186:105535.

14.	Dubinski D, Won SY, Mattes I, et al. Frailty in cerebellar ischemic stroke-The significance of temporal muscle thickness. Frontiers in neurology. 2023;14:1193685.

15.	Nagano A, Shimizu A, Maeda K, et al. Predictive Value of Temporal Muscle Thickness for Sarcopenia after Acute Stroke in Older Patients. Nutrients. 2022;14(23).

16.	Tutal Gürsoy G, Görgülü Ü, Tengirşenk Z, Bektas H. The relationship between temporal muscle thickness and triglyceride glucose index in sarcopenia with mortality and third-month functional outcomes in patients after acute stroke. Medicine. 2023;102(44):e35886.

17.	Katsuki M, Suzuki Y, Kunitoki K, et al. Temporal Muscle as an Indicator of Sarcopenia is Independently Associated with Hunt and Kosnik Grade on Admission and the Modified Rankin Scale Score at 6 Months of Patients with Subarachnoid Hemorrhage Treated by Endovascular Coiling. World neurosurgery. 2020;137:e526-e34.

18.	Lin Y-H, Chung C-T, Chen C-H, et al. Association of temporalis muscle thickness with functional outcomes in patients undergoing endovascular thrombectomy. European Journal of Radiology. 2023;163:110808.

19.	Chung SJ, Kim YJ, Yoo HS, et al. Temporalis Muscle Thickness as an Indicator of Sarcopenia Is Associated With Long-term Motor Outcomes in Parkinson’s Disease. The Journals of Gerontology: Series A. 2021;76(12):2242-8.

20.	Peball M, Valent D, Grossauer A, et al. Temporal Muscle Thickness Correlates with Sarcopenia in Parkinson's Disease. Journal of Parkinson's disease. 2023;13(3):341-50.

21.	Vinciguerra C, Toriello A, Nardone V, et al. Temporal muscle thickness and survival in patients with amyotrophic lateral sclerosis. Neurological research. 2022;44(11):1006-10.

22.	Dubinski D, Won SY, Behmanesh B, et al. Significance of Temporal Muscle Thickness in Chronic Subdural Hematoma. Journal of clinical medicine. 2022;11(21).

23.	Steindl A, Leitner J, Schwarz M, et al. Sarcopenia in neurological patients: standard values for temporal muscle thickness and muscle strength evaluation. Journal of clinical medicine. 2020;9(5):1272.

24.	Bonm AV, Menghini A, Drolet CE, Graber JJ. Temporalis muscle thickness predicts early relapse and short survival in primary CNS lymphoma. Neuro-Oncology Practice. 2023;10(2):162-8.

25.	Cho J, Park M, Moon W-J, Han S-H, Moon Y. Sarcopenia in patients with dementia: correlation of temporalis muscle thickness with appendicular muscle mass. Neurol Sci. 2022;43(5):3089-95.

26.	Maskos A, Schmidbauer ML, Kunst S, et al. Diagnostic Utility of Temporal Muscle Thickness as a Monitoring Tool for Muscle Wasting in Neurocritical Care. Nutrients. 2022;14(21):4498.

27.	Korhonen TK, Arponen O, Steinruecke M, et al. Reduced temporal muscle thickness predicts shorter survival in patients undergoing chronic subdural haematoma drainage. J Cachexia Sarcopenia Muscle. 2024;15(4):1441-50.

# Supplemental Methods

## 2.1. Inter-reader variability in segmenting temporalis muscles

Two readers (both with 3 years of experience in medical imaging segmentation methods) were trained by a musculoskeletal radiologist. The musculoskeletal radiologist’s segmentation was used as the “gold standard” to assess inter-reader performance. These trained readers were blinded to the participants’ risk factors or any other clinical information. The readers manually segmented bilateral temporalis muscles in 619 ADNI 1/GO (Reader-1: n=310, Reader-2: n=309) and 100 ADNI 2 (Reader-1: n=65, Reader-2: n=66) brain MRI examinations. To facilitate and expedite temporalis muscle segmentation, a semi-automated software was developed on the ImageJ platform (version 2.1.0, National Institutes of Health; http://imagej.net), enabling rapid and reliable segmentation of bilateral temporalis muscles.

The variability between different readers was evaluated using the Dice Similarity Coefficient (DSC), which was calculated between each reader and a musculoskeletal radiologist. We tested reproducibility on 30 cases, comparing Reader-1’s and Reader-2’s segmentations to the radiologist’s reference segmentation. Upon testing a set of 30 images, the segmentations made by Reader-1 and Reader-2 yielded DSC values of 0.94 and 0.96, respectively, when compared to the segmentations made by the musculoskeletal radiologist.

## 2.2. MRI acquisition and temporalis measurement

Temporalis muscles were segmented bilaterally on axial T1-weighted brain MRIs at the level of orbital roof landmark.^1-3^ Manual segmentation was performed on 750 ADNI participants using ImageJ (NIH, version 2.1.0), with a semi-automated pipeline allowing rapid and standardized processing. The two readers were blinded to clinical data. Segmentation process and inter-reader reproducibility details are provided above. A 2D U-Net model with a ResNet-50 backbone pretrained on ImageNet was trained to segment the temporalis muscle, using 80% of manually segmented ADNI images as ground truth. The model was subsequently validated on the remaining 20% of ADNI images and then externally validated on 278 randomly selected Visit 5 ARIC MRIs. Performance was quantified using the DSC, and the final temporalis muscle masks used in all downstream radiomic, and cross-sectional area analyses were generated by the U-Net model. Compared to manual human segmentation, the model achieved a DSC of 0.91 in the ADNI test set (n=150) and 0.76 in ARIC (n=278), indicating moderate-to-good agreement in a fully independent dataset.

## 2.3. Radiomic dimensionality reduction and least absolute shrinkage and selection operator (LASSO)–penalized Cox proportional hazards modeling

Five-fold cross-validation was used to select the optimal regularization parameter (λ) for the LASSO-penalized Cox model. As shown in **eFigure 1**, model performance increased with greater shrinkage, peaked, and declined at higher penalization values, demonstrating a conventional bias–variance pattern. The λ in each cohort was therefore used for all subsequent radiomic feature selection steps.

**eFigure 1**. The figure shows the mean 5-fold cross-validated concordance index (C-index) across the range of tested regularization parameters (λ). Model performance varied modestly across λ values, with the highest mean C-index observed at the upper end of the penalization range, indicating that stronger shrinkage improved model generalizability in this cohort. The λ yielding the maximal cross-validated C-index was selected for radiomic modeling in each cohort.

To assess the robustness of LASSO-selected radiomic features, stability selection was performed using 100 subsampled datasets (80% of participants per resample). **eFigure 2** reports the coefficient estimates and selection probabilities for each retained radiomic feature with a non-zero coefficient at the optimal λ in each dataset. Across the 93 candidate radiomic features, ADNI and ARIC selected 33 and 43 features, respectively, with 23 features overlapping (Jaccard index = 0.43) (**eFigure 3**). This degree of overlap indicates moderate-to-strong cross-cohort reproducibility of the LASSO-derived radiomic signature.^4,5^

**eFigure 2**. Stability selection probabilities for radiomic features retained in the LASSO-penalized Cox model. Each bar represents the proportion of 100 subsampled datasets (80% of participants per resample) in which a radiomic feature had a non-zero coefficient under the optimal regularization parameter of each dataset. Of the 93 total radiomic features, 33 were retained in ADNI, and 43 in ARIC, with 23 features overlapping, (Jaccard index = 0.43), indicating robust cross-cohort reproducibility of the penalized feature selection process.

**eFigure 3**. Cross-cohort stability of LASSO-selected radiomic features in ADNI and ARIC. Heatmap showing whether each radiomic feature (rows) was selected by the L1-penalized Cox model in the ADNI or ARIC cohort (columns). Features selected within a cohort appear in dark blue (value = 1), and unselected features appear in light blue (value = 0). Of 93 candidate features, 33 were selected in ADNI and 43 in ARIC, with 23 features overlapping across both cohorts (Jaccard index = 0.43). The cross-cohort overlap demonstrates moderate reproducibility of the radiomic signature despite differences in scanners, demographic composition, and cohort structure.^4,5^

Proportional hazards assumptions were evaluated for the radiomic composite Cox model using Schoenfeld residual–based tests (Kaplan-Meier and rank variants) in both cohorts. All covariates met proportional hazards assumptions except MMSE in ADNI, which showed significant non-proportionality in both test formulations (**eTable 1**). We conducted sensitivity analyses using models allowing a time-varying MMSE coefficient (MMSE × log(time)). This interaction term was statistically significant in both models (*p*<0.005), confirming time-varying effect for MMSE. In these models, estimated associations between temporalis CSA (1.14 [1.01, 1.30]) and composite radiomic score (1.18 [1.01, 1.37]) and incident dementia were qualitatively similar to the primary results.

**eTable 1**. Tests of the proportional hazards assumption for the composite Cox model in ADNI. Schoenfeld residual–based χ² tests (Kaplan-Meier [KM] and rank variants) were used to assess proportionality for each covariate. All variables demonstrated no evidence of violation (*p* > 0.05) except MMSE, which showed significant deviation from proportional hazards.

| **Variable** | **Test Type** | **χ² Statistic** | ***p*-value** | **−log₂(*p*)** |
| --- | --- | --- | --- | --- |
| Temporalis Muscle Radiomic Score | KM | 1.71 | 0.19 | 2.39 |
|  | rank | 1.43 | 0.23 | 2.1 |
| Age | KM | 1.27 | 0.26 | 1.94 |
|  | rank | 1.17 | 0.28 | 1.84 |
| Female vs Male | KM | 0.09 | 0.77 | 0.38 |
|  | rank | 0.27 | 0.60 | 0.73 |
| Non-Hispanic White vs Non-Hispanic Black | KM | 0.54 | 0.46 | 1.11 |
|  | rank | 0.17 | 0.68 | 0.56 |
| Body Mass Index | KM | 0.54 | 0.46 | 1.12 |
|  | rank | 0.28 | 0.60 | 0.75 |
| *APOE*-ε4 Carrier | KM | 0.03 | 0.85 | 0.23 |
|  | rank | 0.06 | 0.80 | 0.32 |
| MMSE | KM | 19.68 | <0.005 | 16.74 |
|  | rank | 19.2 | <0.005 | 16.37 |

**eFigure 4.** Directed acyclic graph. SPPB was only available in ARIC.

# Supplemental Results

**eFigure 5.** Subgroup analysis of Cox proportional hazards models for incident dementia among participants in ADNI and ARIC. Hazard ratios (HRs) for temporalis muscle cross-sectional area (CSA) represent the risk of incident dementia per standard deviation decrease in CSA. HRs for the temporalis muscle radiomic score represent the risk of incident dementia per standard deviation increase in score. Ninety-three radiomic features were used to derive a single radiomic score per participant using a Least Absolute Shrinkage and Selection Operator regression model, which was trained independently in each subgroup with five cross-fold validation. Models were adjusted for baseline age, sex, race (non-Hispanic White vs non-Hispanic Black), *APOE*-ε4 carrier status, body mass index, Mini-Mental State Examination, and in ARIC, Short Physical Performance Battery. Each model did not adjust for the respective covariate that the cohort was being stratified by.

**eFigure 6.** Kaplan-Meier curves depict dementia-free survival according to temporalis muscle cross-sectional area (top panels) and composition/radiomic score (bottom panels) in the Alzheimer’s Disease Neuroimaging Initiative (ADNI; left) and the Atherosclerosis Risk in Communities (ARIC; right) cohorts. Curves represent estimated survival at the cohort mean and at +1 standard deviation (SD) of each temporalis muscle measure. Hazard ratios (HRs) and 95% CIs were derived from multivariable Cox proportional hazards models adjusted for age, sex, race (non-Hispanic White vs non-Hispanic Black), *APOE*-ε4 carrier status, body mass index, Mini-Mental State Examination, and in ARIC, Short Physical Performance Battery. In ADNI, lower temporalis muscle cross-sectional area was associated with higher dementia risk, whereas this association was not observed in ARIC. In contrast, higher temporalis muscle radiomic scores were consistently associated with increased dementia risk in both cohorts.

**eTable 2.** Multivariate Cox-proportional hazards models and their concordance indices (C-index) modeling risk of incident dementia with and without temporalis cross-sectional area (CSA) and radiomic score included as predictors within the Alzheimer’s Disease Neuroimaging Initiative.

**A) ADNI**

|  | **Without temporalis CSA predictor** | **With temporalis CSA predictor** |
| --- | --- | --- |
| Cox proportional hazards C-index | 0.72 [0.70, 0.76] | 0.73 [0.70, 0.76] |
| C-index comparison *p*-value | 0.87 | |
|  | **Without temporalis radiomic score predictor** | **With temporalis radiomic score predictor** |
| Cox proportional hazards C-index | 0.72 [0.70, 0.76] | 0.74 [0.71, 0.77] |
| C-index comparison *p*-value | **0.032** | |

**B) ARIC**

|  | **Without temporalis CSA predictor** | **With temporalis CSA predictor** |
| --- | --- | --- |
| Cox proportional hazards C-index | 0.68 [0.61, 0.74] | 0.68 [0.61, 0.74] |
| C-index comparison *p*-value | 0.61 | |
|  | **Without temporalis radiomic score predictor** | **With temporalis radiomic score predictor** |
| Cox proportional hazards C-index | 0.68 [0.61, 0.74] | 0.70 [0.64, 0.76] |
| C-index comparison *p*-value | 0.17 | |

**eTable 3.** Analysis of Cox proportional hazards models for incident dementia among participants in ADNI and ARIC who did not experience dementia within the first two years of follow-up. Hazard ratios (HRs) for temporalis muscle cross-sectional area (CSA) represent the risk of incident dementia per standard deviation decrease in CSA. HRs for the temporalis muscle radiomic score represent the risk of incident dementia per standard deviation increase in score. Ninety-three radiomic features were used to derive a single radiomic score per participant using a Least Absolute Shrinkage and Selection Operator regression model, which was trained independently in each subgroup with five cross-fold validation. Models were adjusted for baseline age, sex, race (non-Hispanic White vs non-Hispanic Black), *APOE*-ε4 carrier status, body mass index, Mini-Mental State Examination, and in ARIC, Short Physical Performance Battery.

| **Predictor (Z-scored)** | **ADNI** | | **ARIC** | |
| --- | --- | --- | --- | --- |
|  | Hazard Ratio [95% CI] | *p*-value | Hazard Ratio [95% CI] | *p*-value |
| Temporalis Muscle CSA | 1.17 [1.02, 1.34] | **0.024** | 1.06 [0.79, 1.41] | 0.71 |
| Temporalis Muscle Radiomic Score | 1.30 [1.06, 1.59] | **0.013** | 1.61 [1.29, 2.01] | **<0.005** |

**eTable 4.** Analysis of Fine-Gray subdistribution hazards models for incident dementia among participants in ADNI and ARIC censoring all-cause mortality as a competing event. Subdistribution hazard ratios (sHRs) for temporalis muscle cross-sectional area (CSA) represent the risk of incident dementia per standard deviation decrease in CSA. sHRs for the temporalis muscle radiomic score represent the risk of incident dementia per standard deviation increase in score. Ninety-three radiomic features were used to derive a single radiomic score per participant using a Least Absolute Shrinkage and Selection Operator regression model, which was trained independently in each subgroup with five cross-fold validation. Models were adjusted for baseline age, sex, race (non-Hispanic White vs non-Hispanic Black), *APOE*-ε4 carrier status, body mass index, Mini-Mental State Examination, and in ARIC, Short Physical Performance Battery.

| **Predictor (Z-scored)** | **ADNI** | | **ARIC** | |
| --- | --- | --- | --- | --- |
|  | sHR [95% CI] | *p*-value | sHR [95% CI] | *p*-value |
| Temporalis Muscle CSA | 1.17 [1.02, 1.34] | **0.024** | 1.04 [0.78, 1.40] | 0.78 |
| Temporalis Muscle Radiomic Score | 1.30 [1.06, 1.59] | **0.013** | 1.58 [1.27, 1.98] | **<0.005** |

**eTable 5.** Number of participants in ARIC who experienced baseline (visit 5) or incident (visits 6-9) competing neurological events.

| **Neurological event** | Baseline (Visit 5) | Incident (Visits 6-9) |
| --- | --- | --- |
| Stroke/cerebrovascular accident | 40 (7.52%) | 37 (6.95%) |
| Head injury | 77 (14.47%) | 29 (5.45%) |
| Parkinson's disease | 9 (1.69%) | 6 (1.13%) |
| Brain tumor | 0 (0.00%) | 5 (0.94%) |

**eTable 6.** Analysis of Fine-Gray subdistribution hazards models for incident dementia among participants in ARIC censoring neurological outcomes (stroke/cerebrovascular accident, head injury, Parkinson’s disease, brain tumor) and all-cause mortality as competing events. Subdistribution hazard ratios (sHRs) for temporalis muscle cross-sectional area (CSA) represent the risk of incident dementia per standard deviation decrease in CSA. sHRs for the temporalis muscle radiomic score represent the risk of incident dementia per standard deviation increase in score. Ninety-three radiomic features were used to derive a single radiomic score per participant using a Least Absolute Shrinkage and Selection Operator regression model, which was trained independently in each subgroup with five cross-fold validation. Models were adjusted for baseline age, sex, race (non-Hispanic White vs non-Hispanic Black), *APOE*-ε4 carrier status, body mass index, Mini-Mental State Examination, and Short Physical Performance Battery.

| **Predictor (Z-scored)** | **ARIC** | |
| --- | --- | --- |
|  | sHR [95% CI] | *p*-value |
| Temporalis Muscle CSA | 1.04 [0.78, 1.40] | 0.77 |
| Temporalis Muscle Radiomic Score | 1.58 [1.27, 1.98] | **<0.005** |

**eTable 7.** Analysis of Cox proportional hazards models for incident dementia among participants in ARIC who during follow-up visits or at baseline never experienced competing neurological events (stroke/cerebrovascular accident [CVA], head injury, Parkinson’s disease, brain tumor). Hazard ratios (HRs) for temporalis muscle cross-sectional area (CSA) represent the risk of incident dementia per standard deviation decrease in CSA. HRs for the temporalis muscle radiomic score represent the risk of incident dementia per standard deviation increase in score. Ninety-three radiomic features were used to derive a single radiomic score per participant using a Least Absolute Shrinkage and Selection Operator regression model, which was trained independently in each subgroup with five cross-fold validation. Models were adjusted for baseline age, sex, race (non-Hispanic White vs non-Hispanic Black), *APOE*-ε4 carrier status, body mass index, Mini-Mental State Examination, and Short Physical Performance Battery.

| **Excluding those with** | Temporalis Muscle CSA (Z-scored) | | Temporalis Muscle Radiomic Score (Z-scored) | |
| --- | --- | --- | --- | --- |
|  | Hazard Ratio [95% CI] | *p*-value | Hazard Ratio [95% CI] | *p*-value |
| Excluding those with incident stroke/CVA | 1.04 [0.78, 1.40] | 0.77 | 1.70 [1.34, 2.17] | **<0.005** |
| Excluding those with baseline and incident stroke/CVA | 1.17 [0.88, 1.57] | 0.28 | 1.76 [1.35, 2.29] | **<0.005** |
| Excluding those with incident head injury | 1.05 [0.79, 1.41] | 0.73 | 1.68 [1.33, 2.13] | **<0.005** |
| Excluding those with baseline and incident head injury | 1.00 [0.75, 1.34] | 1.00 | 1.75 [1.33, 2.29] | **<0.005** |
| Excluding those with incident Parkinson’s disease | 1.02 [0.76, 1.36] | 0.90 | 1.58 [1.27, 1.97] | **<0.005** |
| Excluding those with baseline and incident Parkinson’s disease | 1.02 [0.76, 1.36] | 0.90 | 1.57 [1.25, 1.96] | **<0.005** |
| Excluding those with incident brain tumor | 1.05 [0.79, 1.41] | 0.73 | 1.61 [1.29, 2.00] | **<0.005** |
| Excluding those with baseline and incident brain tumor | 1.05 [0.79, 1.41] | 0.73 | 1.61 [1.29, 2.00] | **<0.005** |

**eTable 8.** Dental and oral health factors in participants in ARIC collected at visit 4, which is 13-17 years before visit 5 (considered as the baseline in the rest of the analysis).

| **Dental/oral health factor** | **Visit 4** |
| --- | --- |
| Lost any natural teeth | 74 (13.91%) |
| Have any false teeth | 261 (49.06%) |
| Ever had a root canal done | 231 (43.42%) |
| Ever had a dental implant | 10 (1.88%) |

**eTable 9.** Analysis of Cox proportional hazards models for incident dementia among participants in ARIC, adjusting for dental/oral health factors. Hazard ratios (HRs) for temporalis muscle cross-sectional area (CSA) represent the risk of incident dementia per standard deviation decrease in CSA. HRs for the temporalis muscle radiomic score represent the risk of incident dementia per standard deviation increase in score. Ninety-three radiomic features were used to derive a single radiomic score per participant using a Least Absolute Shrinkage and Selection Operator regression model, which was trained independently in each subgroup with five cross-fold validation. Models were adjusted for baseline age, sex, race (non-Hispanic White vs non-Hispanic Black), *APOE*-ε4 carrier status, body mass index, Mini-Mental State Examination, Short Physical Performance Battery, and the specified dental/oral health predictor.

| **Adjusting for dental/oral health variable** | Temporalis Muscle CSA (Z-scored) | | Temporalis Muscle Radiomic Score (Z-scored) | |
| --- | --- | --- | --- | --- |
|  | Hazard Ratio [95% CI] | *p*-value | Hazard Ratio [95% CI] | *p*-value |
| Additionally adjusting for loss of natural teeth | 1.07 [0.80, 1.42] | 0.67 | 1.61 [1.29, 2.01] | **<0.005** |
| Additionally adjusting for false teeth | 1.02 [0.76, 1.36] | 0.91 | 1.60 [1.28, 1.99] | **<0.005** |
| Additionally adjusting for root canal | 1.05 [0.79, 1.41] | 0.72 | 1.63 [1.30, 2.03] | **<0.005** |
| Additionally adjusting for dental implant | 1.07 [0.80, 1.42] | 0.67 | 1.62 [1.30, 2.02] | **<0.005** |

# Supplemental References

1.	Leitner J, Pelster S, Schöpf V, et al. High correlation of temporal muscle thickness with lumbar skeletal muscle cross-sectional area in patients with brain metastases. *PloS one*. 2018;13(11):e0207849.

2.	Steindl A, Leitner J, Schwarz M, et al. Sarcopenia in neurological patients: standard values for temporal muscle thickness and muscle strength evaluation. *Journal of clinical medicine*. 2020;9(5):1272.

3.	Cho J, Park M, Moon W-J, Han S-H, Moon Y. Sarcopenia in patients with dementia: correlation of temporalis muscle thickness with appendicular muscle mass. *Neurological Sciences*. 2022/05/01 2022;43(5):3089-3095. doi:10.1007/s10072-021-05728-8

4.	Bologna M, Corino VDA, Montin E, et al. Assessment of Stability and Discrimination Capacity of Radiomic Features on Apparent Diffusion Coefficient Images. *J Digit Imaging*. Dec 2018;31(6):879-894. doi:10.1007/s10278-018-0092-9

5.	Wong LM, Ai QYH, Zhang R, Mo F, King AD. Radiomics for Discrimination between Early-Stage Nasopharyngeal Carcinoma and Benign Hyperplasia with Stable Feature Selection on MRI. *Cancers (Basel)*. Jul 14 2022;14(14)doi:10.3390/cancers14143433
